# $\mathscr{P}\mathscr{T}$-symmetric KdV solutions and their algebraic extension with zero-width resonances

**DOI:** 10.1038/s41598-024-65432-3

**Published:** 2024-07-03

**Authors:** Kumar Abhinav, Aradhya Shukla, Prasanta K. Panigrahi

**Affiliations:** 1 Centre for Theoretical Physics and Natural Philosophy, Nakhonsawan Studiorum for Advanced Studies, Mahidol University, Nakhonsawan, 60130 Thailand; 2 Department of Physics, Institute of Applied Sciences and Humanities, GLA University, Mathura, Uttar Pradesh 281406 India; 3 Indian Institute of Science Education and Research Kolkata, Mohanpur, West Bengal 741246 India; 4 Center for Quantum Science and Technology, Siksha o Anusandhan University, Bhubaneswar, Odisha 751030 India

**Keywords:** Nonlinear phenomena, Theoretical physics, Quantum mechanics

## Abstract

A class of complex breather and soliton solutions to both KdV and mKdV equations are identified with a Pöschl-Teller type $\mathscr{P}\mathscr{T}$-symmetric potential. However, these solutions represent only the unbroken-$\mathscr{P}\mathscr{T}$ phase owing to their isospectrality to an infinite potential well in the complex plane having real spectra. To obtain the broken-$\mathscr{P}\mathscr{T}$ phase, an extension of the potential satisfying the $sl\left( 2,\mathbb {R}\right)$ potential algebra is mandatory that additionally supports non-trivial zero-width resonances.

## Introduction

The $\mathscr{P}\mathscr{T}$-symmetric Schrödinger system is characterized by potentials with parity-even real and parity-odd imaginary parts^1–3^. As a result the Hamiltonian *H*, which is now symmetric under combined parity ($\mathscr{P}$) and time-reversal ($\mathscr{T}$) operations, is no longer Hermitian. Yet, contradicting the notion of quantum mechanical Hilbert space, such non-Hermitian Hamiltonians ($H^\dagger \ne H$) still possess real eigenvalues^4^,

1 $$\begin{aligned} H\psi =E\psi ,\quad E\in \mathbb {R}, \end{aligned}$$

for a certain range of parameters of the system. Such a situation physically depicts the balancing out of gain and loss due to the imaginary part of the Hamiltonian, as realized in the optical analogs of $\mathscr{P}\mathscr{T}$-symmetric systems^5^. Such a scenario mandates a ‘norm’ for these systems which is an extension of the von Neumann-Dirac case of standard quantum mechanics^4,6–8^. Beyond a parameter threshold, however, the characteristic spectra transform into complex-conjugate pairs^4,9,10^ representing the spontaneous breaking of $\mathscr{P}\mathscr{T}$-symmetry^5,11–13^. The broken phase may further contain zero-width resonances^14–16^, which are well-resolved eigenstates, having real energies^17^.

Non-linear systems possessing $\mathscr{P}\mathscr{T}$-symmetry have been the subject of many recent investigations^18–23^. The effect of non-linearity on $\mathscr{P}\mathscr{T}$-symmetric potentials leads to localized and stable wave packets known as self-trapped modes^24,25^. Highly stable and localized unique solutions, or solitons, have been observed in $\mathscr{P}\mathscr{T}$-symmetric optical lattice^26^. Therein, the intrinsic non-linearity of the system plays an important role in their stability^27^. Moreover, a system with defocusing (positive) non-linearity that possesses $\mathscr{P}\mathscr{T}$-symmetry due to an odd gain-loss distribution can have real spectra^28^. Its $\mathscr{P}\mathscr{T}$-symmetry further remains unbreakable for arbitrarily large strength of the gain-loss term^29^. $\mathscr{P}\mathscr{T}$-symmetry is also responsible for integrability of the non-local non-linear Schrödinger equation^30^ that supports localized solutions. Soliton, kink, and other localized structures are further observed in non-local sine-Gordon^31^ and Kundu-nonlocal-Schrödinger^32^ systems recently, with the latter possessing unique asymptotic behavior^33^.

The Korteweg-de Vries (KdV) equation^35,36^ is one of the most renowned systems that finds application in various physical phenomena^34^. In particular, complex KdV solitons^34^ have been found to respect $\mathscr{P}\mathscr{T}$-symmetry^37,38^. Notably, one of the Lax pairs of the KdV equation constitutes the Schrödinger eigenvalue equation with the KdV solution as the Schrödinger potential^39^. This explains the stability and robustness of the KdV solitons. In particular, the Pöschl-Teller potential^40^ is also a soliton solution to the KdV system. This potential is reflectionless, a property that naturally explains the solitons passing through each other without scattering^41^. Inverse scattering transformation provides further justification for this connection^42–44^.

The reflectionless nature of the Pöschl-Teller potential is due to its isospectrality to the free particle^45^, *i. e.*, they share an identical energy spectrum. In the framework of supersymmetric quantum mechanics (SUSY-QM)^47,48^, two isospectral potentials $$V_\pm (x)=W^2(x)\pm W'(x)$$, with a common superpotential *W*(*x*) share the spectra except for the ground state: $$E_{n+1}^-=E_n^+$$^46^. Consequently, even a trivially solvable potential can be connected to a non-trivial one and vice-versa, providing a convenient algebraic way to solve a complicated Schrödinger equation.

Given a Schrödinger potential that is a solution to the KdV system, the corresponding superpotential satisfies the modified KdV (mKdV) equation^37,49^. This is because the respective solutions to these two equations are related through the Miura transformation: $u = v^2 \pm v_x$^50,51^. Moreover, since there are two distinct mKdV equations with solutions connected as $v \rightarrow iv$, there is another class of KdV solutions with functional form: $u = -v^2 \pm iv_x$^50,52^. This suggests the complexification of the space for a better understanding of the complex potential. Given the potential function is also a $\mathscr{P}\mathscr{T}$-symmetric KdV solution, this further suggests connections to the broken and unbroken phases of $\mathscr{P}\mathscr{T}$. Both periodic and localized solutions of KdV systems have already been related to the complex Pöschl-Teller potential^37,52^. It is, therefore, of deep interest to investigate if the complex $\mathscr{P}\mathscr{T}$-symmetric KdV solutions^38^ are isospectral to the free particle. Two such complex solutions of interest are breathers, which are particular periodic solutions^53^, and solitons given their physical importance. Further, the possible free particle connection to the broken $\mathscr{P}\mathscr{T}$ phase of a complexified Pöschl-Teller potential is also of importance.

In the present work, a class of complex breathers and solitons for the KdV system has been identified as $\mathscr{P}\mathscr{T}$-symmetric potentials under a general parameterization. A sub-class of these solutions further corresponds to superpotentials that solve the mKdV system as complex breathers and solitons through the Miura transformations. The general class of solutions is isospectral to a free particle in a 1-dimensional box embedded in the complex plane. Given the width of the box is real, isospectrality demands the relevant $\mathscr{P}\mathscr{T}$-symmetry to be unbroken. With a further extension of the ground state that conforms to an underlying $sl(2,\mathbb {R})$ potential algebra, a more general $\mathscr{P}\mathscr{T}$-symmetric system is obtained. The latter possesses a spontaneously $\mathscr{P}\mathscr{T}$-broken sector with complex-conjugate spectra including zero-width resonances. It is to be noted that the complex potential in the broken-$\mathscr{P}\mathscr{T}$ phase does not satisfy the KdV equation and its connection with the free particle is not possible.

The paper has been organized as follows. The next section demonstrates the complex breather and soliton solutions for the KdV system. Identifying the general form of these solutions as a $\mathscr{P}\mathscr{T}$-symmetric potential isospectral to an infinite potential well in a complex plane physically justifies their structures. This further yields a set of complex breather and soliton solutions to the mKdV system in terms of superpotentials. The section thereafter provides the generalized version of the $\mathscr{P}\mathscr{T}$-symmetric potential that further supports a $\mathscr{P}\mathscr{T}$-broken phase with the possibility of zero-width resonances. The latter property mandates an additional $sl\left( 2,\mathbb {R}\right)$ algebraic structure of the potential. The last section concludes the paper along with the prospects for future works.

## Complex KdV breathers and solitons

The focusing and defocussing-type KdV equations are respectively given as,

2 $$\begin{aligned} u_t \pm 6\,u\, u_X + u_{XXX} =0. \end{aligned}$$

The defocussing case supports propagating solutions described in terms of a time parameter *t*, defined through $\tau = v\,t+x_0$^54^. For simplicity, we take $x_0 =0$ in order to retain the symmetric nature of potential at $t=0$. The KdV equation is known to possess breather^53,55^ and complex soliton^37,38,52^ solutions. Among the prior, the Akhmediev breathers^56–58^ are periodic in space and localized in time whereas Ma breathers^59^ have the opposite behavior. A valid periodic solution to the KdV system has the form,

3 $$\begin{aligned} u^B_1(X,t)=-a^2+2a^2\csc ^2(aX+ 4a^3\,t+i\eta ), \end{aligned}$$

where the $a,\,\eta \in \mathbb {R}$. Another parameterization,

4 $$\begin{aligned} u^B_2(X,t)=-a^2+2a^2\csc ^2(aX-2a^3\,t+i\eta ), \end{aligned}$$

also satisfies the same equation. Both $u^B_{1,2}(X,t)$ are periodic in space and time which are the characteristics of a general breather^55^ that is periodic in nature. It is worth mentioning that breathers have been observed experimentally in Bose-Einstein condensates^60^ and optical waveguides^61^. Recently, a similar kind of solution has also been found for focusing-type KdV equation $u_t+6uu_X+u_{XXX}=0$ that represents a complex soliton instead^62^, where the time-delay analysis has been performed^38^. This complex soliton can be expressed in two ways,

5 $$\begin{aligned}{} & {} u^S_1(X,t)=-a^2+2a^2\csc ^2(\xi -iaX+i4a^3t),\nonumber \\{} & {} u^S_2(X,t)=-a^2+2a^2\csc ^2(\xi +iaX+i2a^3t), \end{aligned}$$

wherein $$a,\,\xi \in \mathbb {R}$$.

The periodic (breathers) and localized (complex solitons) nature of these two classes of solutions can be understood from their interpretation as potentials to the Schrödinger equation^39^. These solutions (potentials) have a general form,

6 $$\begin{aligned} u(X,t)=u(\xi ,\,\eta )=-a^2+2a^2\csc ^2(\xi +i\eta ), \end{aligned}$$

with suitable choices of the parameters $\xi ,\eta \in \mathbb {R}$ leading to particular breathers and solitons discussed above. This general form is a potential isospectral to the one-dimensional free particle in an infinite box^48^, connected through the relation,

7 $$\begin{aligned} V_\pm (\xi ,\,\eta )=W^2(\xi ,\,\eta )\pm W_{\xi }(\xi ,\,\eta ). \end{aligned}$$

On identifying $V_+(\xi ,\,\eta )$ with $u(\xi ,\,\eta )$, $V_-(\xi ,\,\eta )$ is a constant defining the boxed free particle for a superpotential,

8 $$\begin{aligned} W(\xi ,\,\eta )=-a\cot (\xi +i\eta ). \end{aligned}$$

Then the parameter *a* is simply the ground state momentum of the particle confined in the box of length $L=\pi /a$. However, subtlety arises as the quantum system is defined now in a complex plane spanned by $\left( \xi ,i\eta \right)$. In order to possess a real spectrum the width of the box needs to be aligned with the $\xi$ axis. In that case the ‘free particle’ wave-function $\psi (\xi ,\,\eta )$ is periodic in $\xi$ but localized in $\eta$. This mirrors the breather and soliton behaviors observed in terms of particular KdV solutions obtained above through suitable substitutions of $\xi$ or $\eta$ respectively.

Further, Eq. 7 exactly mimics the Miura transformations^39^:

9 $$\begin{aligned} u(X,t)=v_1^2\pm v_{1,X}=v_2^2\pm iv_{2,X}, \end{aligned}$$

that relates a KdV solution to a set of mKdV solutions related as $v_2(X,t)=iv_1(X,t)$^37,52^. Since *u*(*X*, *t*) is identified with $V_+(\xi ,\,\eta )$, $W(\xi ,\,\eta )$ solves the mKdV equations^50,52^,

10 $$\begin{aligned} v_{1,t} \pm 6v^2_1 v_{1,X}+v_{1,XXX} =0. \end{aligned}$$

In particular, for $\xi =aX-2a^3t$ with $\eta$ being a parameter $W(\xi ,\,\eta )$ is a complex breather for the defocussing mKdV system $v_{1,t}-6v^2_1 v_{1,X}+v_{1,XXX} =0$. Alternatively, if $\xi$ serves as a parameter and $\eta =aX+2a^3t$ then $W(\xi ,\,\eta )$ is a complex soliton for the focusing mKdV system $v_{1,t}+6v^2_1 v_{1,X}+v_{1,XXX} =0$. However, either for $\xi =aX+4a^3t$ or for $\eta =-aX+4a^3t$, the Miura transformation does not possess any solution $W(\xi ,\,\eta )$ that solves the mKdV equation. So there are two distinct classes of KdV solutions emerging from the isospectral potential.

**Table 1:** Conditions for obtaining KdV and mKdV solutions.

Class	$\xi$	$\eta$	$V_+$	*W*
I	$aX+4a^3t$	Parameter	Breather for Defocussing KdV	None
	Parameter	$-aX+4a^3t$	Soliton for Focusing KdV	None
II	$aX-2a^3t$	Parameter	Breather for Defocussing KdV	Breather for Defocussing mKdV
	Parameter	$aX+2a^3t$	Soliton for Focusing KdV	Soliton for Focusing mKdV

**Figure 1:**
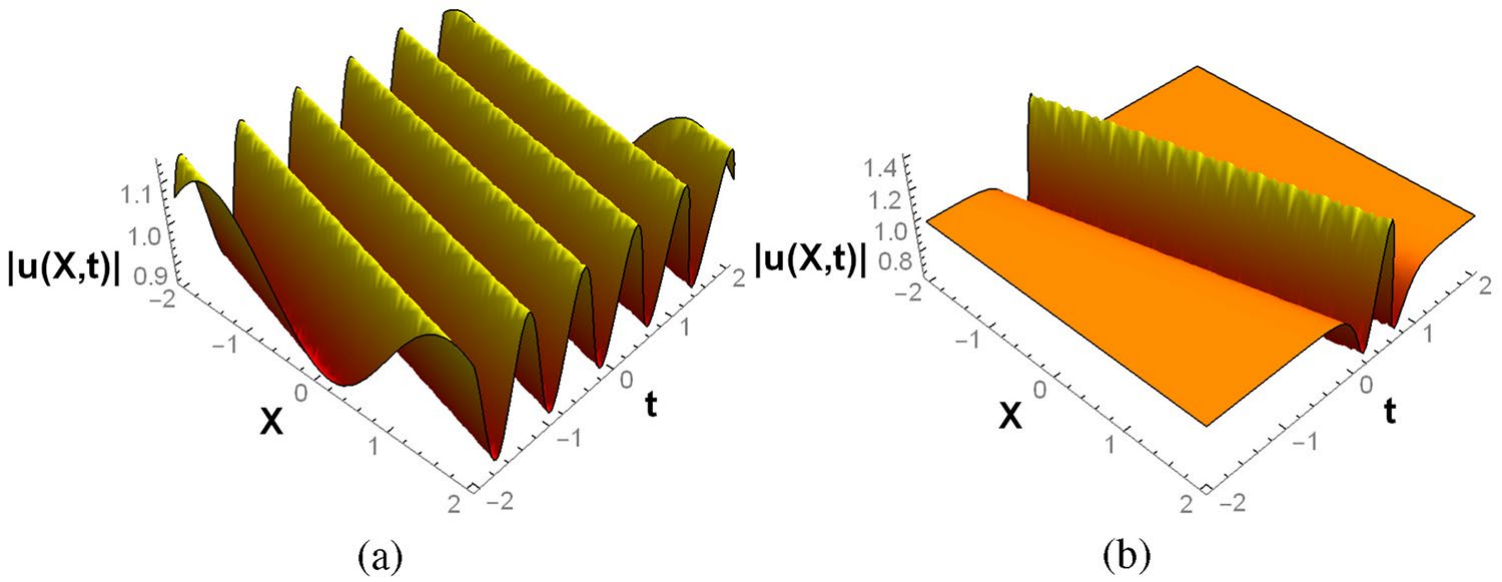
KdV solutions of class I: (**a**) Complex breather for $\xi =aX+4a^3t$ with $\eta =2$ and (**b**) complex soliton for $\eta =-aX+4a^3t$ with $\xi =2$. In both cases $a=1$.

**Figure 2:**
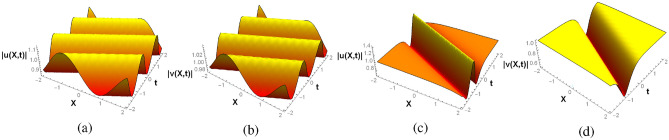
Solutions of class II: (**a**) Complex KdV and (**b**) complex mKdV breathers for $\xi =aX-2a^3t$ with $\eta =2$. (**c**) Complex KdV and (**d**) complex mKdV solitons for $\eta =aX+2a^3t$ with $\xi =2$. In all cases $a=1$.

Table 1 and Figs. 1 and 2 depict some examples of these two classes of periodic and localized complex solutions. The complex breathers show periodic variation whereas the complex solitons are localized in the real space-time. Indeed, on expanding the solution in Eq. 8 in terms of $\xi$ and $\eta$, one obtains a structure similar in form to the generalized KdV breather obtained by Zaworski^55^ yet distinct in individual harmonic and hyperbolic components. This new class of KdV (and mKdV) solutions, with breathers and solitons being particular cases, owes its structure to the isospectrality with the complex free particle. Their complex nature is further consistent with the general Miura transformation and essentially follows from the attributed $\mathscr{P}\mathscr{T}$-symmetry of the corresponding Schrödinger system, to be explained next. As can be seen from Figs. 1a, 2a, b the complex breathers for both KdV and mKdV systems display similar behavior. However, the complex KdV solitons for both the classes are of W-type and of grey-bright nature as shown in Figs. 1b and 2c. On the other hand, the mKdV counterpart of class II in Fig. 2d is grey in nature. These structures are consistent with the Miura transformations connecting them.

**Figure 3:**
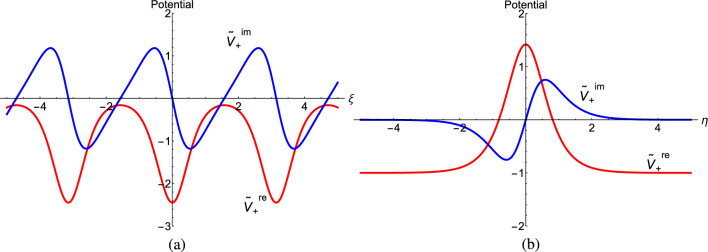
$\mathscr{P}\mathscr{T}$-symmetric potential: (**a**) $\mathbb {R}e(\tilde{V}_+(x))$ and $\mathbb {I}m(\tilde{V}_+(x))$ vs. $\xi$ and (**b**) $\mathbb {R}e(\tilde{V}_+(x))$ and $\mathbb {I}m(\tilde{V}_+(x))$ vs. $\eta$.

The reality of the quantum spectrum, a result of the one-dimensional infinite box being along the real $\xi$ axis, is physically motivated by the inherent $\mathscr{P}\mathscr{T}$-symmetry^1–3^ of the potential $V_+(\xi ,\,\eta )$. This is evident as its real part is symmetric in both $\xi$ and $\eta$ whereas the imaginary part is anti-symmetric in both, as shown in Fig. 3. As iterated before, such non-Hermitian Schrödinger systems support real spectrum in the $\mathscr{P}\mathscr{T}$-symmetric phase. Otherwise, the spectrum splits up into complex conjugate pairs representing the ‘broken’ phase. Herein, the ambiguity in defining parity in 2-dimensions needs to be considered. We choose $(\xi \rightarrow -\xi ,\,\eta \rightarrow \eta )$ over $(\xi \rightarrow \xi ,\,\eta \rightarrow -\eta )$ on physical grounds since the spectrum is shared by the superpartner potentials. Then the symmetric phase of $V_+(\xi ,\,\eta )$ ensures real spectra of $V_-(\xi ,\,\eta )$. This implies that the width of the potential well *L* must be real and thus $\xi$ is the dynamical coordinate. The symmetric phase of $V_+(\xi ,\,\eta )$ is further assured by the uniqueness of $W(\xi ,\,\eta )$^63^, a fact that independently mandates the 1-d potential well to be aligned along $\xi$.

The proposed complex breather and soliton solutions of the KdV system, as well as those for the mKdV system, all correspond to the $\mathscr{P}\mathscr{T}$-symmetric phase of a complex Schrödinger potential. In this phase, the potential is isospectral to an infinite potential well in the complex plane that has a real spectrum. The breathers correspond to the real coordinate $\xi$ of the quantum system whereas the solitons are related to the imaginary one $\eta$. This is reflected in the respective periodic and localized natures of $V_+(\xi ,\,\eta )$ in terms of $\xi$ and $\eta$, along with its $\mathscr{P}\mathscr{T}$-symmetry. In the next section, we generalize the superpotential through parametric extensions incorporating both broken and unbroken phases of $\mathscr{P}\mathscr{T}$. The spectrum in the $\mathscr{P}\mathscr{T}$-broken phase further supports a subset containing real energies, called zero-width resonances, given certain algebraic criteria are met.

## Generalized complex potential: accessing the $\mathscr{P}\mathscr{T}$-broken phase

For a general complex potential $V(\xi , \eta )$ on the $\xi$-$\eta$ plane, the Schrödinger eigenvalue equation is given as,

11 $$\begin{aligned} \Big (\frac{\partial ^2}{\partial \xi ^2} - \frac{\partial ^2}{\partial \eta ^2} - V(\xi ,\eta ) \Big )\, \Psi (\xi ,\eta ) = - k^2 \, \Psi (\xi ,\eta ), \end{aligned}$$

with energy $E=k^2$. Considering a particular solution of the form: $\Psi (\xi ,\eta ) = \Phi (\xi ) \, e^{i{\tilde{k}}\eta }$, with a complex momentum ${\tilde{k}}$, results in,

12 $$\begin{aligned} \frac{1}{\Phi (\xi )}\Big (\frac{\partial ^2}{\partial \xi ^2} - V(\xi ,\eta ) \Big )\Phi (\xi ) + k^2 = -{\tilde{k}}^2. \end{aligned}$$

On taking $V=0$ in the case of the infinite well and imposing the boundary conditions, $\Psi (\xi _1, \eta _1) = 0=\Psi (\xi _2, \eta _2)$, the particle is confined to move along the real axis $\xi$ that results in a real spectrum. The orthogonal direction $i\eta$ corresponds to an imaginary momentum affecting an exponential decay of the eigenstates. Consequently, the isospectral $\mathscr{P}\mathscr{T}$-symmetric potential $V_+(\xi ,\eta )$ must be confined to its $\mathscr{P}\mathscr{T}$-symmetric phase. The corresponding eigenfunctions,

13 $$\begin{aligned}{} & {} \psi ^+_n (\xi ,\eta ) \propto (n+1)\,\Bigg [\cos \{(n+2)\xi \}\cosh \{(n+2)\eta \}-i\sin \{(n+2)\xi \}\sinh \{(n+2)\eta \}\Bigg ]\nonumber \\{} & {} \qquad \qquad \quad -\frac{\cos \{(n+1)\xi \}\sinh \{(n+1)\eta \}- i\sin \{(n+1)\xi \}\cosh \{(n+1)\eta \} }{\big (\sin \xi \,\cosh \eta - i \,\cos \xi \, \sinh \eta \big )}, \end{aligned}$$

further ensure this as they are $\mathscr{P}\mathscr{T}$-symmetric in $\xi$. Additionally, a unique superpotential ${\tilde{W}}(\xi ,\eta )$ generates this whole spectrum, which is a defining characteristic of unbroken $\mathscr{P}\mathscr{T}$-symmetry^63^. This is true unless any additional symmetry is involved^64^ and this phase supports a vanishing ‘current’^6^. With appropriate boundary restrictions, these eigenfunctions are periodic in the real direction whereas they exponentially decay along the imaginary one, as was the case for the infinite potential well. If alternate boundary conditions can change this decaying nature, it should lead to a non-zero associated current^6^ and thereby to the $\mathscr{P}\mathscr{T}$-broken phase.

Just like ${\tilde{V}}_+(\xi ,\eta )$, its eigenstates $\psi _n^+(\xi ,\eta )$ are also $\mathscr{P}\mathscr{T}$-symmetric under the alternate parity choice: $\xi \rightarrow \xi ,\,\eta \rightarrow -\eta$ that we had avoided on physical grounds. Evidently, within no range of the present parameters, the superpartners can display spontaneous breaking of $\mathscr{P}\mathscr{T}$-symmetry. Naturally, there is a more general form of ${\tilde{V}}_\pm (\xi ,\eta )$ which reduces to that in Eq. (6) when parametric conditions for $\mathscr{P}\mathscr{T}$-symmetry are imposed^63^. A simple extension could be when $\tilde{W}(\xi ,\eta )$ is generalized to *two* superpotentials $\tilde{W}_1(\xi ,\eta )=-\left( a\pm i\gamma \right) \cot \left( \xi +i\eta \right)$. Herein, $a,\alpha ,\gamma \in \mathbb {R}$ with $\xi =\alpha x_{re}$ and $\eta =\alpha x_{im}$. This equivalently amounts to a parametrically more generalized ground state. The corresponding generalized supersymmetric potentials,

14 $$\begin{aligned} \tilde{V}^1_-(x)=(a\pm i\gamma )(a\pm i\gamma -\alpha )\csc ^2\left( \xi +i\eta \right) , \end{aligned}$$

up to a constant energy, are connected to the free particle under certain parametric conditions^65–67^. Notably, this generalization of the parameters no longer satisfies the KdV and thereby the mKdV equations. This is because moving away from the real spectrum essentially means that the reflectionless nature of the corresponding Schrödinger potential, essential for localized non-linear solutions^41^, is lost. Although, the generalized potentials still maintain shapes similar to Figs. 1 and 2 and are now shape-invariant^48,68^ under the transformation $a\rightarrow a+\alpha$. There are indeed two superpotentials, which are necessary for $\mathscr{P}\mathscr{T}$-symmetry breaking^63^. The two potentials themselves, however, converge to a *unique* one on imposition of the condition: $\gamma (2a-\alpha )=0$ that makes them $\mathscr{P}\mathscr{T}$-symmetric. The $\mathscr{P}\mathscr{T}$-symmetric phase itself further requires $\gamma =0$ with real energies $E^{1,\textrm{s}}_n=(a+n\alpha )^2$ that can be obtained through shape invariance^63^. In particular, $V^1_-(x)$ represents a free particle for $\alpha =a$. The broken phase has $a=\alpha /2$ with a complex-conjugate paired spectrum,

15 $$\begin{aligned} E^{1,\textrm{b}}_n=\left( n\alpha +\frac{\alpha }{2}\pm i\gamma \right) ^2. \end{aligned}$$

This spectrum is always complex and does not allow for exceptional points since $n=-1/2$ is not possible. It is worth noting that the isospectrality to any real system is not possible in this sector. Further confirmation comes from the eigenstates of the system,

16 $$\begin{aligned} \psi _n^{1,\textrm{b}}(y)\propto & {} \left( -\frac{d}{dy}+\tilde{W}_1(y)\right) ^n\left( \sin (y)\right) ^{a+n\alpha \pm i\gamma },\nonumber \\ y= & {} \xi +i\eta , \end{aligned}$$

as they cannot result in a vanishing Wronskian unless $\gamma =0$ (symmetric phase), despite yielding a non-vanishing current^6^, .

In order to obtain non-trivial zero-width resonances, we further generalize the parameters of the superpotential as

17 $$\begin{aligned} \tilde{W}_2(\xi ,\eta )=-a\cot \left( \xi +i\eta \right) +(b\pm i\lambda )\tan \left( \xi +i\eta \right) ,\quad a,b,\lambda\in & {} \mathbb {R}. \end{aligned}$$

Now there is no similarity left to the KdV (and mKdV) solutions obtained before, even in terms of the shape of the potentials. The $\mathscr{P}\mathscr{T}$-symmetry of the corresponding potential is imposed through the condition $\lambda (2b-\alpha )=0$. The symmetric phase further requires $\lambda =0$, leading to a unique superpotential. The resultant potential and the subsequent spectrum are:

18 $$\begin{aligned}{} & {} \tilde{V}^{2,\textrm{s}}_-(\xi ,\eta )=a(a-1)\csc ^2\left( \xi +i\eta \right) +b(b-\alpha )\sec ^2\left( \xi +i\eta \right) ,\nonumber \\{} & {} E^{2,\textrm{s}}_n=(a+b+2n\alpha )^2, \end{aligned}$$

the latter obtained through shape-invariance shifts $a\rightarrow a+\alpha ,\quad b\rightarrow b+\alpha$. This potential is the trigonometric analog to the Pöschl-Teller II potential. The broken phase requires $b=\alpha /2$, which maintains two superpotentials, still leading to a unique potential and subsequent spectrum:

19 $$\begin{aligned}{} & {} \tilde{V}^{2,\textrm{b}}_-(\xi ,\eta )=a(a-1)\csc ^2\left( \xi +i\eta \right) -\left( \lambda ^2+\frac{1}{4}\right) \sec ^2\left( \xi +i\eta \right) ,\nonumber \\{} & {} E^{2,\textrm{b}}_n=\left( a+\frac{\alpha }{2}+2n\alpha \pm i\gamma \right) ^2. \end{aligned}$$

Zero-width resonances are now possible for parametric exceptional points $a=-2n\alpha -\alpha /2$. This is consistent with the fact that *a* must represent free-particle ground state momentum in the symmetric phase. The eigenstates in the broken sector,

20 $$\begin{aligned} \psi _n^{2,\textrm{b}}(y) \propto \left( -\frac{d}{dy}+\tilde{W}_2(x)\right) ^n\left( \sin (y)\right) ^{a+n\alpha }\left( \cos (y)\right) ^{\frac{\alpha }{2}+n\alpha \pm i\lambda }, \end{aligned}$$

also correspond to a non-vanishing current, and more interestingly, to vanishing Wronskians for $a=-2n\alpha -\alpha /2$. Here, it is worth noting that the choice of the ground state, or equivalently the choice of the superpotential, plays a key role in capturing the $\mathscr{P}\mathscr{T}$-broken phase. The disconnection with the KdV equation is demonstrated again by the loss of isospectrality with the free particle.

To obtain non-trivial zero-width resonances the $\tan (\xi +i\eta )$ term in $\tilde{W}_2(\xi ,\eta )$ is necessary. Its importance follows from the $sl\left( 2,\mathbb {R}\right)$ algebraic structure^69^ obeyed by the isospectral potentials (For real potentials with sinusoidal functions, it is *SO*(3). For potentials containing hyperbolic functions, it is *SO*(2, 1) for real ones and $sl\left( 2,\mathbb {C}\right)$ for $\mathscr{P}\mathscr{T}$-symmetric ones^64^). Though $\tilde{V}^{2,\textrm{b}}_-(\xi ,\eta )$ does not satisfy this algebra directly, it can be re-arranged as a trigonometric analog of the Pöschl-Teller potential that does^64^:

21 $$\begin{aligned}{} & {} \frac{1}{4}\left( \Lambda -\Gamma \right) \left( \Lambda -\Gamma -\alpha \right) \csc ^2(y)+\frac{1}{4}\left( \Lambda +\Gamma \right) \left( \Lambda +\Gamma +\alpha \right) \sec ^2(y)\nonumber \\{} & {} \qquad \qquad =\left[ \Lambda ^2+\Gamma \left( \Gamma +\alpha \right) \right] \csc ^2(2y)-\Lambda \left( 2\Gamma +\alpha \right) \csc (2y)\cot (2y),\quad \Gamma ,\Lambda \in \mathbb {R}. \end{aligned}$$

Herein the coefficients should be consistent with those of $\tilde{V}^{2,\textrm{b}}_-(y)$. The potential form on the RHS of Eq. 21 satisfies the $sl\left( 2,\mathbb {R}\right)$ potential algebra and corresponds to a superpotential $\bar{W}(y)=\Gamma \cot (2y)-\Lambda \csc (2y)$. In terms of the parameters of the algebra, the potential takes the form,

22 $$\begin{aligned} V_m(z)=\left( \frac{1}{4}-m^2\right) \frac{dF(z)}{dz}+2m\frac{dG(z)}{dz}+G(z)^2,\quad z=2, \end{aligned}$$

that enables the identification,

23 $$\begin{aligned} \bar{W}(z)={\left\{ \begin{array}{ll} \textrm{either}\quad \left( \mp m-\frac{1}{2}\right) F(z)\pm G(z)\\ \textrm{or}\qquad \quad \left( \pm \beta -\frac{1}{2}\right) F(z)\pm \frac{m}{\beta }G(z). \end{array}\right. } \end{aligned}$$

The two functions $F(z)=\cot (z)$ and $G(z)=\beta \csc (z)$ are a particular $sl\left( 2,\mathbb {R}\right)$ representation that need to satisfy,

24 $$\begin{aligned} \frac{dF}{dz}=-1-F^2\quad \textrm{and}\quad \frac{dG}{dz}=-FG. \end{aligned}$$

The complete algebra is represented as (The $sl\left( 2,\mathbb {C}\right)$ counterpart of this was obtained in references^64,69^),

25 $$\begin{aligned}{} & {} \left[ J_0,\,J_\pm \right] =\pm J_\pm ,\quad \left[ J_+,\,J_-\right] =2J_0;\nonumber \\{} & {} J_0=\frac{\partial }{\partial _\varphi },\nonumber \\{} & {} J_\pm =e^{\pm \varphi }\left[ \pm \frac{\partial }{\partial z}+\left( -\frac{\partial }{\partial \varphi }\mp \frac{1}{2}\right) F(z)+G(z)\right] , \end{aligned}$$

with the Casimir $J^2=J_0^2-J_0+J_+J_-$. The corresponding spectrum is,

26 $$\begin{aligned}{} & {} J^2\vert k,m\rangle =k(k-1)\vert k,m\rangle ,\quad J_0\vert k,m\rangle =m\vert k,m\rangle ,\nonumber \\{} & {} m=-k,-k+1,\cdots ,k. \end{aligned}$$

It is clear that $\tilde{V}^{1,\textrm{b}}_-(\xi ,\eta )$ corresponds to a reduced $sl\left( 2,\mathbb {R}\right)$ representation with $F(y)=\cot (y)$ and $G(y)=0$. Indeed the underlying potential algebra for a potential of $\csc ^2(\xi +i\eta )$-type is *SU*(1, 1)^70^, which is isomorphic to $sl\left( 2,\mathbb {R}\right)$. For $\mathscr{P}\mathscr{T}$-symmetry all the coefficients in Eq. 21 must be real as $y\rightarrow -y$ under $\mathscr{P}\mathscr{T}$, which is consistent with the fact that $\Gamma ,\Lambda \in \mathbb {R}$. This considerably restricts the parameters $a(\Gamma ,\Lambda )$, $b(\Gamma ,\lambda )$ and $\lambda (\Gamma ,\lambda )$ and it follows that $b,\lambda \ne 0$ for $a\in \mathbb {R}$. In other words, since *a* is the real momentum of the 1-D infinite well, a sufficiently general $\mathscr{P}\mathscr{T}$-symmetric structure that leads to non-trivial zero-width resonances demands that $\tilde{W}_2(x)$ carries the $\tan (\xi +i\eta )$ term. A simpler alternative could have been to extend $\tilde{W}(x)$ to:

27 $$\begin{aligned} \tilde{W}_3(\xi ,\eta )=-a\cot \left( \xi +i\eta \right) +\mathcal{B}\csc \left( \xi +i\eta \right) ,\quad a\in \mathbb {R}, \end{aligned}$$

which directly provides a set of non-trivial *F* and *G* satisfying $sl\left( 2,\mathbb {R}\right)$. However, the existence of a $\mathscr{P}\mathscr{T}$-broken phase requires more than $\mathcal{B}\in \mathbb {C}$ since the corresponding spectrum $E^{3,b}_n=(a+n\alpha )^2$ does not feature $\mathcal{B}$. This would correspond to a more ‘drastic’ change in parameterization than that for $\tilde{W}_2(x)$ *e. g.* $a\in \mathbb {C}$. The potential algebra for this system (Pöschl-Teller II potential) is *SO*(2, 2)^71^ which is not directly connected to $sl(2,\mathbb {R})$. Consequently, the importance of a non-trivial $sl\left( 2,\mathbb {R}\right)$ (or $sl\left( 2,\mathbb {C}\right)$ in the hyperbolic case) representation to obtain zero-width resonances in the $\mathscr{P}\mathscr{T}$-broken phase is further emphasized.

## Conclusion

In conclusion, it is shown that the $\mathscr{P}\mathscr{T}$-symmetric complex extension to the Pöschl-Teller potential encompasses novel complex breather and soliton solutions *u* of the KdV system. This happens when the $\mathscr{P}\mathscr{T}$-symmetry is preserved (symmetric phase) as the system maintains isospectrality to a free particle in a 1-d box that is aligned to the real axis of the complex plane. As a result, the corresponding spectra remain real. Furthermore, the generalization of the superpotential leads to shape-invariant partner potentials. This provides a natural connection, through two forms of Miura transformations $u = v^2 \pm v_x$ and $u=-v^2 \pm iv_x$, to solutions *v* of the mKdV equation. Finally, a properly generalized construction of the ground state with complex energy can access the $\mathscr{P}\mathscr{T}$-broken sector of the system. The $sl(2,\mathbb {R})$ potential algebra must be satisfied to achieve this sector, which further supports the states with zero-width resonances. Therefore, a physical realization of these resonances in the broken-$\mathscr{P}\mathscr{T}$ phase is obtained from a manifest algebraic perspective.

In the future, the KdV hierarchy of the isospectral potentials connected to the free particle within the framework of $\mathscr{P}\mathscr{T}$-symmetry will be worth exploring. Their connection to multi-soliton solutions^54^ is also worth venturing. Since the complex KdV system is also related to the Levi-Civita equation^72,73^, it would be interesting to study the latter in the view of $\mathscr{P}\mathscr{T}$-symmetry. In addition, the connection of $\mathscr{P}\mathscr{T}$-breaking to the corresponding potential algebra needs further investigation. Presently, the Berry phase correlated with the complex solution in the $\mathscr{P}\mathscr{T}$-broken phase is under investigation and will be reported elsewhere.

## Data Availability

All data that support the findings in this study are available in the article. Additional information is available from the corresponding author upon request.
